# Dielectric Properties of Shrinkage-Free Poly(2-Oxazoline) Networks from Renewable Resources †

**DOI:** 10.3390/polym13081263

**Published:** 2021-04-13

**Authors:** Fabio Blaschke, Philipp Marx, Stefan Hirner, Inge Mühlbacher, Karin Wewerka, Frank Wiesbrock

**Affiliations:** 1 Polymer Competence Center Leoben GmbH, Roseggerstrasse 12, 8700 Leoben, Austria; fabio.blaschke@pccl.at (F.B.); philipp.marx@pccl.at (P.M.); stefan.hirner@pccl.at (S.H.); inge.muehlbacher@pccl.at (I.M.); 2 Institute for Chemistry and Technology of Materials, Graz University of Technology, NAWI Graz, Stremayrgasse 9, 8010 Graz, Austria; 3 Chair of Chemistry of Polymeric Materials, Montanuniversitaet Leoben, Otto-Gloeckel-Str. 2, 8700 Leoben, Austria; 4 Center for Electron Microscopy, Institute for Electron Microscopy and Nanoanalysis, Graz University of Technology, Steyrergasse 17, 8010 Graz, Austria; karin.wewerka@felmi-zfe.at

**Keywords:** nanodielectrics, volumetric expansion, renewable resources, poly(2-oxazoline), polyamide 12, dual-cure mechanism, thermal conductivity

## Abstract

In the course of this study, the dielectric and physicochemical properties of poly(2-oxazoline) (POx) networks from renewable resources were compared with those of fossil-based polyamide 12 (PA 12) networks. POx was synthesized by the energy-efficient, microwave-assisted copolymerization of 2-oxazoline monomers, which were derived from fatty acids of coconut and castor oil. For the preparation of composites, aluminum nitride nanoparticles n-AlN and microparticles μ-AlN as well as hexagonal boron nitride BN submicroparticles were used. Additionally, 0, 15, or 30 wt.% of a spiroorthoester (SOE) were added as an expanding monomer aiming to reduce the formation of shrinkage-related defects. For the crosslinking of the polymers and the SOE as well as the double ring-opening reaction of the SOE, a thermally triggered dual-cure system was developed. The fully-cured blends and composites containing SOEs exhibited lower densities than their fully-cured SOE-free analogues, which was indicative of a lower extent of shrinkage (or even volumetric expansion) during the curing reaction, which is referred to as relative expansion RE. The RE amounted to values in the range of 0.46 to 2.48 for PA 12-based samples and 1.39 to 7.50 vol.% for POx-based samples. At 40 Hz, the “green” POx networks show low loss factors, which are competitive to those of the fossil-based PA 12.

## 1. Introduction

Due to the trend of miniaturization and the inherently increasing power density of electronic devices, the next generation of polymer-based insulating materials must meet higher standards, in particular increased thermal conductivity and increased dielectric performance. Since both the thermal conductivity and the dielectric properties of polymers can be influenced by reinforcement with nano-scaled inorganic particles, the preparation of the so-called nanodielectrics has gained increased attention during the last two decades. For the preparation of nanodielectrics, various electrically insulating inorganic oxide nanoparticles such as silica SiO_2_, alumina Al_2_O_3_, zinc oxide ZnO, titanium oxide TiO_2_, etc. have been used [1,2,3,4]. One characteristic of nanodielectrics is the large polymer–particle interface, which often results in unique and unexpected properties [5]. Hence, nanocomposites with improved dielectric performance, e.g., reduced permittivity and loss factor [6,7] or increased breakdown strength [8], can be prepared. 

Due to the increasing heat development in electronic devices, sufficient thermal stability and heat dissipation are key requirements for the next generation of dielectrics. Since polymers show poor thermal conductivities in the range of 0.1 to 0.3 W·m^−1^K^−1^, the addition of micro- and nano-scaled fillers with intrinsic high thermal conductivity is the most common strategy to provide sufficient heat dissipation. In addition to the above-mentioned oxide fillers, also ceramic fillers such as aluminum nitride AlN (110–260 W·m^−1^K^−1^) [9], hexagonal boron nitride BN (up to 390 W·m^−1^K^−1^) [10], and silicon carbide SiC (130–160 W·m^−1^K^−1^) [11] are employed due to their high thermal conductivity. The thermal conductivity of composites depends on various factors such the thermal conductivity of the filler, the size and shape of the filler, the distribution of the filler, and the thermal resistance at the particle–polymer interface [12].

Another challenge during the preparation of thermoset-based dielectrics is the volumetric shrinkage during the curing reaction due to the formation of covalent bonds, which are shorter than the Van der Waals distances between the unreacted monomers. This curing shrinkage induces mechanical stress in the polymer network and can result in the formation of microfractures and delamination [13,14]. Hence, shrinkage reduction is of great interest for the preparation of defect-free dielectrics. In addition to the compounding with high amounts of fillers and the usage of prepolymers, ring-opening polymerizations can be used to reduce shrinkage. 

**Figure 1:**
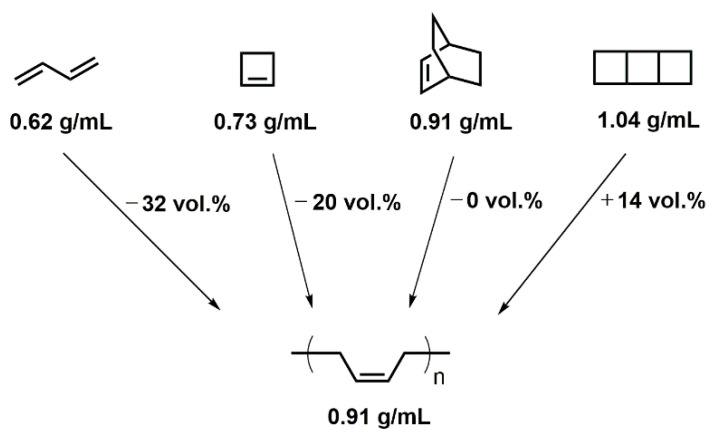
Schematic overview of the volumetric changes during the (hypothetical) polymerization of 1,3-butadiene, cyclobutene, bicyclo[2.2.2]oct-2-ene, and tricyclo[4.2.0.0^2,5^]octane upon the formation of poly(butadiene). Reprinted from reference [17] with permission of the authors.

Cyclic molecules have higher densities than their acyclic analogues (Figure 1); hence, the shrinkage during ring-opening polymerizations is commonly lower than that during chain- and step-growth polymerizations [15,16]. Correspondingly, the volumetric change correlates with the number of rings, which are opened during the polymerization. This effect can be demonstrated by the (theoretical) polymerization of 1,3-butadiene (no ring present), cyclobutene (1 ring present), bicyclo[2.2.2]oct-2-ene (2 rings present), and tricyclo[4.2.0.0^2,5^]octane (3 rings present) upon the formation of poly(butadiene) (Figure 1) [17]. In the 1970s, bicyclic oxygen-containing molecules such as spiroorthoesters, spiroorthocarbonates, and bicyclic carbonates were found to polymerize by cationic double ring-opening reaction upon volumetric expansion [18]. Hence, this class of monomers is referred to as expanding monomers and can be used as additive to reduce the curing shrinkage during conventional polymerizations [19,20,21].

Resins/polymer networks such as crosslinked polyethylene (XLPE), poly(amide)s, poly(imide)s, epoxy resins, silicone resins, and phenol–formaldehyde resins, which are commonly used for the preparation of dielectric materials, are obtained from petrochemicals. Sustainability and environmental aspects are an additional important driving force for the development of the next generation of polymer-based dielectrics. Numerous congeners of the class of (co-)poly(2-oxazoline)s, which can be classified as “pseudo”-polyamides, can be synthesized from renewable resources, and, hence, offer an alternative to fossil-based polyamides. The two 2-oxazoline monomers used in this study, namely 2-nonyl-2-oxazoline and 2-dec-9’-enyl-2-oxazoline, can be synthesized from the fatty acids decanoic and undec-10-enoic acid, which are derived from coconut and castor oil, respectively. Notably, the petrochemical ethanol amine is also produced in animal tissue as the biogenic amine of serine [22]. Precedent studies revealed that the permittivity, loss factor and conductivity of poly(2-oxazoline) (POx) networks is in the same range as that of polyamides [23,24].

**Figure 2:**
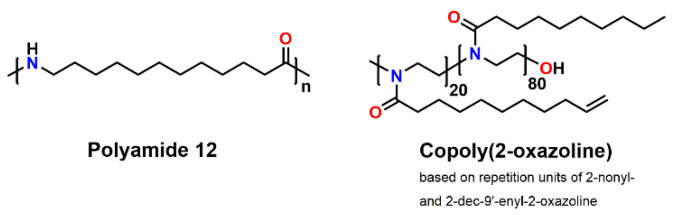
Chemical structure of polyamide 12 (PA 12) and the poly(2-oxazoline) (POx)copolymer.

This study aimed at the preparation of POx- and polyamide 12 (PA 12)-based composites with increased thermal conductivity and reduced shrinkage during the curing reaction. In order to reduce volumetric shrinkage and the occurrence of shrinkage-related defects, both types of polymers were crosslinked with different amounts of an expanding monomer, namely a spiroorthoester (SOE). The crosslinking of the (“pseudo”-)polyamide and the SOE as well as the ring-opening reaction of the SOE were accomplished in a thermally induced dual-cure reaction. The dielectric properties such as permittivity and loss factor as well as the thermal diffusivity of the materials were measured, with special respect to the comparison of the “green” POx with the fossil-based PA 12 (Figure 2). In particular, the influence of different fillers and filler combinations of AlN nano- and microparticles as well as BN submicroparticles and the degree of shrinkage/expansion on the materials’ properties was investigated. 

## 2. Materials and Methods

### 2.1. Materials

Allyl glycidyl ether, boron trifluoride diethyl etherate, γ-butyrolactone, dicumyl peroxide, ethanolamine, methyl tosylate, titanium(IV)butoxide, triethylamine, decanoic acid, and undec-10-enoic acid were purchased from Sigma Aldrich (Vienna, Austria). Aluminum(III)nitride (particle size 1.0–15.0 µm), 1-butylpyridin-1-ium hexafluorophosphate(V) were received from ABCR (Karlsruhe, Germany). Aluminum(III)nitride (particle size 50 nm) was purchased from Ionic Liquid Technologies GmbH IoLiTec (Heilbronn, Germany). Hexagonal boron nitride (particle size 70 nm) was purchased from MKNano (Missiauga, Canada). PA 12 (particle size 25–30 µm) was purchased from Goodfellow Limited (Hamburg, Germany). All chemicals were used as received, except for methyl tosylate that was distilled prior to use.

### 2.2. Instrumentation

The microwave-assisted polymerization was performed with the Synthos 3000 Microwave Synthesis Platform from Anton Paar GmbH (Graz, Austria) in screwable Wheaton^®^ glass vials (15 × 46 mm^2^) with a Rotor 64, equipped with silicon carbide storage racks. The temperature was monitored with an IR sensor. All nuclear magnetic resonance NMR measurements were performed with a Bruker Advance III 300 MHz spectrometer (Bruker BioSpin Corporation, Billerica, MA, USA). As solvent, CDCl_3_ with an internal standard of 0.03% trimethylsilane TMS was used. Fourier-transformed infrared FT-IR spectroscopy was performed with an Alpha Fourier-Transform Infrared Spectrometer with attenuated total reflection (ATR) support (Bruker Optics Inc., Billerica, MA, USA). The scan range was from 400 to 4000 cm^−1^, and 48 scans were performed for each measurement. For the determination of the average molar mass of the polymers, size exclusion chromatography (SEC) analyses were performed on a Shimadzu SEC system (Shimadzu Austria, Vienna, Austria) with a Shimadzu LC-20AD pump, a SIL-20ACHT sampler, and an RID202A refractive index detector. A styrene-divinyl benzene copolymer network-based linear XL 5 µm column (PSS-SDV) by Polymer Standards Service, Mainz, Germany) was used. As solvent, tetrahydrofurane (THF) was used. For the preparation of the PA 12 and POx specimens, a Collin P 200 Laboratory Platen Press was used (Dr. Collin GmbH, Ebersberg, Germany). The thermogravimetry (TGA) and differential scanning calorimetry (DSC) measurements were performed with the NETZSCH STA 449C Jupiter device coupled with quadrupole mass spectrometry (Netzsch, Selb, Germany). The temperature range was from 20 to 550 °C with a heating rate of 10 K·min^−1^ under helium atmosphere. The thermal diffusivity of the materials was measured on an LFA 467 Hyperflash system (Netzsch, Selb, Germany) at temperatures of 20, 60, 100, 140, and 180 °C. The samples were coated with a thin graphite layer before the measurement in order to avoid reflection. Dielectric characterization was performed with a SPECTANO 100 from OMICRON electronics GmbH (Klaus, Austria). All samples were characterized in a frequency range from 0.1 to 5 kHz at room temperature. All samples were dried in a vacuum oven at 60 °C and 1 mbar for 72 h prior to the measurements. Scanning electron microscopy (SEM) measurements were performed using a Tescan Vega 3 scanning electron microscope (Kohoutovice, Czech Republic); electron energy levels were set to 20 kV. The zeta potential measurements were conducted on a SurPASS Electrokinetic Analyzer from Anton Paar using an adjustable Gap Cell (Anton Paar GmbH, Graz, Austria). The electrolyte solution was a 0.01 M KCl solution; for titration, 0.05 M HCl and 0.05 M NaOH solutions were used.

### 2.3. Analysis of the Size of the Used AlN and BN Fillers

**Figure 3:**
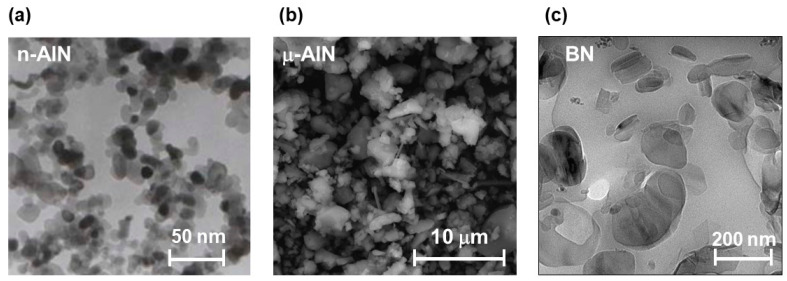
(**a**) TEM image of the nano-sized n-AlN particles. Reprinted with kind permission from IoLiTec—Ionic Liquid Technologies GmbH; all rights reserved. (**b**) SEM image of the micron-sized μ-AlN particles. (**c**) TEM image of the submicron-sized BN particles. Reprinted from reference [23] with kind permission from John Wiley and Sons.

In order to determine the size of the commercially available fillers used in this study, transmission electron microscopy (TEM) and scanning electron microscopy (SEM) measurements were performed (Figure 3). The n-AlN nanoparticles show diameters below 50 nm (Figure 3a), the μ-AlN microparticles show diameters in the range of 1 to 5 μm (Figure 3b), and the BN particles show diameters in the submicrometer range (Figure 3c).

### 2.4. Synthesis of the Monomers

The 2-oxazoline monomers, namely 2-nonyl-2-oxazoline (NonOx) and 2-dec-9’-enyl-2-oxazoline (Dec^=^Ox), were synthesized according to the Henkel patent [25]. The 2-((allyloxy)methyl)-1,4,6-trioxaspiro[4.4]nonane (SOE-allyl) was synthesized according to a literature protocol [26]. 

### 2.5. Synthesis of the Copolymer Poly(2-Nonyl-2-oxazoline)-Stat-Poly(2-Dec-9′-enyl-2-oxazoline) 

The copoly(2-oxazoline) was synthesized by microwave-assisted polymerization. First, 15.81 g of NonOx (80 mmol, 80 equiv.), 4.19 g of Dec^=^Ox (20 mmol, 20 equiv.), and 186.5 mg of methyl tosylate (1.00 mmol, 1 equiv.) were added to 20 mL of acetonitrile. The solution was transferred to dried glass vials, which were placed in silicon carbide plates (with high microwave absorbance) in the Synthos 3000 Microwave Synthesis Platform reactor, and stirred at 140 °C for 2 h. 

The solvent was removed under reduced pressure, and the obtained solid was dried under vacuum using a Schlenk line. After drying, 19.8 g (98%) of the copolymer pNonOx_80_-*stat*-pDec^=^Ox_20_ were obtained as colorless solid.

^1^H-NMR (300 MHz, 298 K, CDCl_3_): δ (ppm) = 0.81 (274H, m), 1.19 (1275H, m), 1.51 (211H, m), 1.97 (45H, m), 2.17 (208H, m), 3.37 (400H, m), 4.88 (40H, m), 5.74 (20H, m). 

^13^C-NMR (75 MHz, 298 K, CDCl_3_): δ (ppm) = 14.1, 22.7, 25.2, 25.4, 29.0, 29.2, 29.4, 29.6, 32.0, 33.0, 33.8, 43.3, 45.3, 114.2, 139.1, 173.2, 173.7. 

IR (ATR): ν (cm^−1^) = 2921, 2852, 1640, 1462, 1430, 1183, 1177, 907, 773, 722. 

GPC: M_w_ = 17.6 kDa; M_n_ = 8.9 kDa; Đ = 1.81.

### 2.6. Preparation of the Polymer Networks and Composites

As polymer matrices, pNonOx_80_-*stat*-pDec^=^Ox_20_ and PA 12 were used. The radical initiator dicumyl peroxide was added in quantities of 5 mol% (pNonOx_80_-*stat*-pDec^=^Ox_20_, which referred to the amount of carbon double bonds present in the polymer) and 2 wt.% (PA 12, referred to the total amount of PA 12), respectively. The SOE-allyl was added with 0, 15 or 30 wt.% (referred to the total amount of polymer). As cationic initiator, 5 wt.% (referred to the amount of SOE-allyl) of 1-butylpyridin-1-ium hexafluorophosphate(V) were added. The overall filler content of the composites was fixed at 40 wt.%, while the following filler combinations were used: 40 wt.% BN, 20/20 wt.% of n-AlN/μ-AlN, and 20/10/10 wt.% of BN/n-AlN/μ-AlN. All mixtures were homogenized by grinding in a mortar. 

For the preparation of the test specimens, the corresponding amounts of the homogenized mixtures were cured in a platen press at 180 °C and 40 bar for 20 min. Therefore, the mixtures were transferred into a steel template with the required geometry, which was placed between two steel plates covered with poly(tetrafluoroethylene) (PTFE)-foil.

## 3. Results

### 3.1. Library Design 

**Figure 4:**
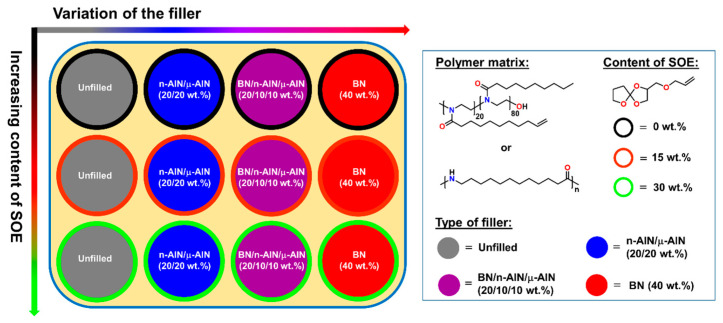
Schematic representation of the material library of the prepared polymer-networks and composites.

For the preparation of the unfilled polymer networks and the composites, two different polymer matrices, namely a self-synthesized POx and commercially available PA 12, were used. In order to reduce the volumetric shrinkage and the formation of shrinkage-related defects during crosslinking of the polymers, SOE-allyl was added as expanding monomer with contents of 0, 15 and 30 wt.%. For the preparation of the corresponding composites, three types of particles, namely nano-sized n-AlN particles, micron-sized μ-AlN particles, and submicron-sized BN particles were used. The filler content of the composites was fixed at 40 wt.%, while three different filler combinations were investigated: 40 wt.% of BN, 20/20 wt.% of n-AlN/μ-AlN, and 20/10/10 wt.% of BN/n-AlN/μ-AlN. Correspondingly, for each type of polymer, a 3 × 4 = 12-membered material library was prepared (Figure 4).

### 3.2. Synthesis of the Monomers and the Copoly(2-Oxazoline)

**Figure 5:**
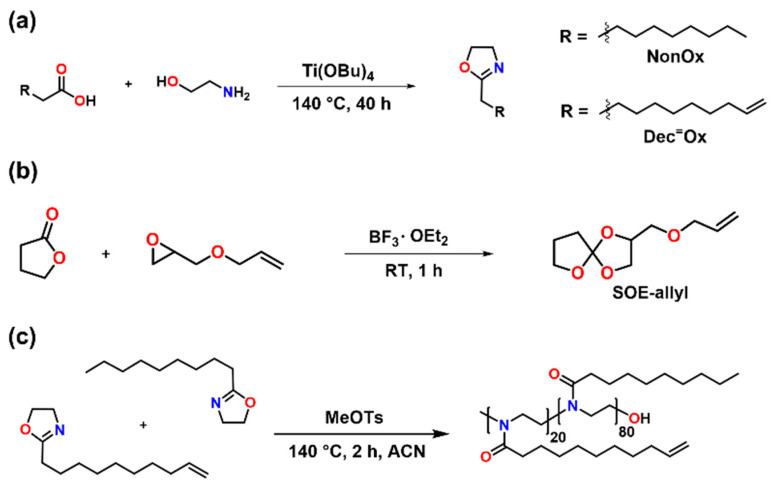
(**a**) Schematic representation of the synthesis of the 2-oxazoline monomers 2-nonyl-2-oxazoline (NonOx) and 2-dec-9’-enyl-2-oxazoline (Dec^=^Ox) from fatty acids and ethanol amine. (**b**) Schematic representation of the synthesis of the spiroorthoester SOE-allyl. (**c**) Microwave-assisted copolymerization of NonOx and Dec^=^Ox.

The monomers 2-nonyl-2-oxazoline (NonOx) and 2-dec-9’-enyl-2-oxazoline (Dec^=^Ox) were synthesized from fatty acids and ethanol amine in a solvent-free reaction according to the Henkel patent (Figure 5a) [25]. The fatty acids, namely decanoic and undec-10-enoic acid, were derived from coconut and castor oil, respectively. As an expanding monomer, an SOE with allyl functionality, namely 2-((allyloxy)methyl)-1,4,6-trioxaspiro[4.4]nonane (SOE-allyl), was synthesized from γ-butyrolactone and allyl glycidyl ether according to a literature protocol (Figure 5b) [26]. 

**Figure 6:**
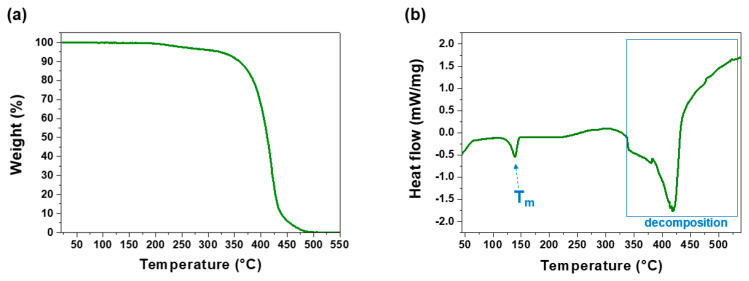
Characterization of the POx poly(2-nonyl-2-oxazoline)_80_-*stat*-poly(2-dec-9′-enyl-2-oxazoline)_20_ by (**a**) thermogravimetry (TGA) and (**b**) differential scanning calorimetry (DSC) measurements.

The microwave-assisted cationic ring-opening polymerization of NonOx and Dec^=^Ox in acetonitrile yielded poly(2-nonyl-2-oxazoline)_80_-*stat*-poly(2-dec-9′-enyl-2-oxazoline)_20_ in quantitative yield (Figure 5c). The purity and composition of the copoly(2-oxazoline) was proven by ^1^H-NMR spectroscopy. Thermogravimetric analysis (Figure 6a) revealed a high-temperature stability of the POx: The copolymer decomposed in one step in the temperature range from 350 to 500 °C. The melting point T_m_ = 138 °C of the polymer was determined by differential scanning calorimetry (Figure 6b).

### 3.3. Crosslinking of the Polymers and Quantification of the Relative Volumetric Expansion

**Figure 7:**
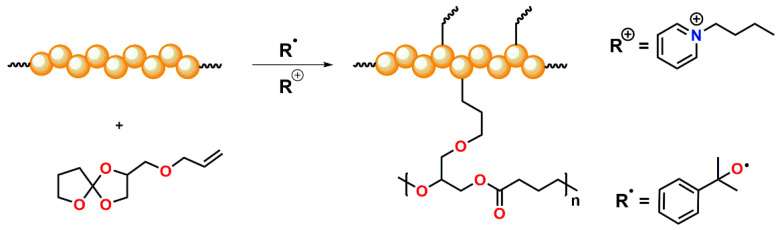
Schematic representation of the thermally triggered dual-cure reaction, comprising the radical-induced crosslinking of the polymers and the allyl group of the SOE as well as the cationic double ring-opening reaction of the SOE groups.

For the crosslinking of PA 12 or the POx (“pseudo”-)polyamide with SOE-allyl, a thermally triggered dual-cure system was developed (Figure 7). The crosslinking of the polymers as well as the covalent attachment of SOE-allyl into the polymer networks involving the C=C-double bond proceeded by radical-mediated reactions using dicumyl peroxide as the radical initiator. For the cationic double ring-opening polymerization of the SOE groups upon volumetric expansion, 1-butylpyridin-1-ium hexafluorophosphate(V) was used as initiator. The corresponding mixtures of polymer, filler, SOE, and initiator system were carefully homogenized and cured in platen press at a temperature of 180 °C and pressure of 40 bar.

For the quantification of the volumetric changes during crosslinking, the density of the cured materials was determined (Table 1 and Table 2). Therefore, the mass and the dimensions of rectangular specimens were measured, and the corresponding density was calculated as quotient of the mass and the volume of the specimens. 

The fully cured blends and composites containing SOEs exhibited lower densities than their fully cured SOE-free analogues, which is indicative of a lower extent of shrinkage (or even volumetric expansion) during the curing reaction, which is referred to as *relative* expansion RE. The RE was calculated was calculated from the densities ρ of the fully cured samples according to Equation (1).

(1) $$\mathrm{RE}\;\left(\%\right)\;=\;\frac{\rho \;(\mathrm{fully}\;\mathrm{cured}\;\mathrm{sample},\;\mathrm{SOE}-\mathrm{free})\;-\;\rho \;(\mathrm{fully}\;\mathrm{cured}\;\mathrm{sample},\;15/30\;\mathrm{wt}.\%-\;\mathrm{SOE})\;}{\rho \;(\mathrm{fully}\;\mathrm{cured}\;\mathrm{sample},\;\mathrm{SOE}-\mathrm{free})}\cdot 100$$

The relative expansion of the POx-based materials is in the range of +1.39% to +4.97% for SOE contents of 15 wt.% and in the range of +4.39% to +7.50% for SOE contents of 30 wt.%, respectively (Table 3). By contrast, the PA 12-based materials show smaller relative expansions RE < 1.0% for SOE contents of 15 wt.%, and REs in the range of 1.41% to 2.48% for SOE contents of 30 wt.% (Table 4).

### 3.4. Thermal Diffusivity of the Polymer Networks and the Composites

**Figure 8:**
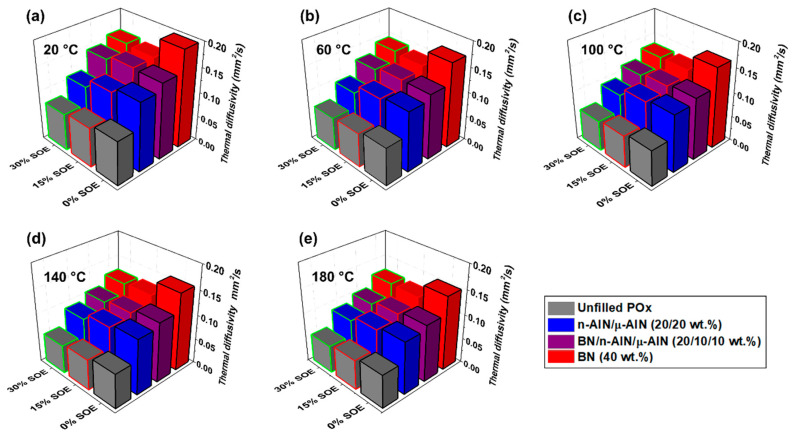
Thermal diffusivity of the POx-based materials at (**a**) 20 °C, (**b**) 60 °C, (**c**) 100 °C, (**d**) 140 °C, and (**e**) 180 °C.

**Figure 9:**
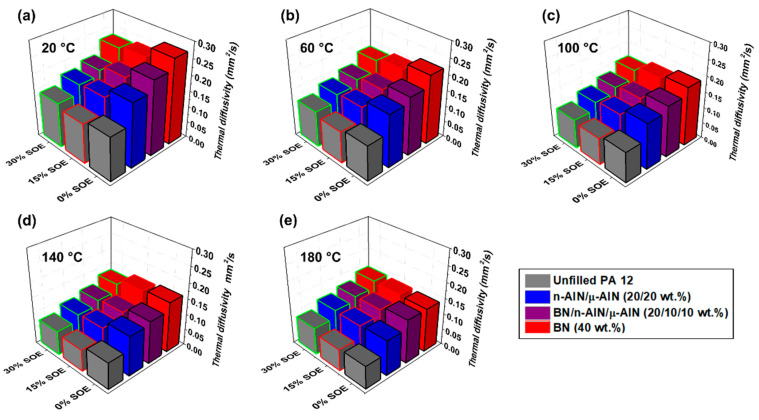
Thermal diffusivity of the PA 12-based materials at (**a**) 20 °C, (**b**) 60 °C, (**c**) 100 °C, (**d**) 140 °C, and (**e**) 180 °C.

The thermal diffusivity of the POx- and PA 12-based composites was measured by light flash analysis (LFA) at temperatures of 20, 60, 100, 140, and 180 °C (Figure 8 and Figure 9). For the thermal diffusivity of both the PA 12- and POx-based materials, general trends can be observed:

(i) The PA 12-based polymer networks exhibit higher thermal diffusivity than the POx-based polymer networks. This is assumed to originate from crystalline domains, the presence of which reduces phonon scattering, which are favorably formed in PA 12 due to hydrogen bonding among adjacent secondary amide groups. By contrast, the “pseudo”-amide POx copolymers contain only tertiary amide groups, between which no hydrogen bonds can be formed (Figure 5c). Additionally, the networks contain long aliphatic side chains, which are likely to hinder the formation of crystalline domains [27]. Complementary zetapotential measurements revealed that the 12 POx/PA 12 resins and composite samples exhibit different isoelectric points, namely in the range of pH = 5.9–8.6/3.9–5.6, which is additionally indicative of the significantly different behavior of the secondary amide in comparison with the tertiary one.

(ii) The assumption of the facilitated formation of crystalline regions in PA 12 is supported by the observation that at high temperatures and inherent higher segmental mobility of the polymers, the values of the thermal diffusivity of both polymer systems converge. For example, at 20 °C, the unfilled PA 12 and POx networks exhibit thermal diffusivities of 0.14 and 0.09 mm·s^−2^; by contrast, at 180 °C, both polymers show a similar thermal diffusivity of 0.073 (PA 12) and 0.068 mm·s^−2^ (POx). In general, the thermal diffusivity of all samples decreases with increasing temperature. 

(iii) By the addition of fillers, the thermal diffusivity is significantly increased. With respect to the used filler system, the thermal diffusivity of the materials increases in the following order: unfilled < n-AlN/μ-AlN < BN/n-AlN/μ-AlN < BN. Correspondingly, the BN composites show the highest thermal diffusivity, which is approximately double as high compared to the unfilled polymers. 

(iv) The thermal diffusivity decreases with increasing content of SOE. This can be explained on the one hand by the higher thermal conductivity of polyamides compared to polyesters and polyethers (during the ring-opening polymerization of the SOE groups polyester-*co*-polyether chains are formed), and on the other by the reduced density of the polymer networks due to volumetric expansion (Table 3 and Table 4).

### 3.5. Dielectric Spectroscopy

**Figure 10:**
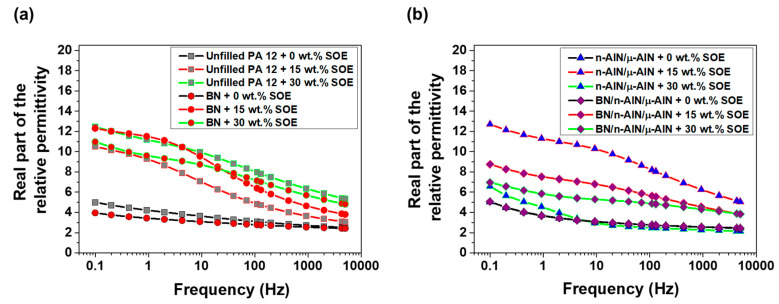
Real part of the relative permittivity of the PA 12-based materials. (**a**) Unfilled PA 12 samples and composites with BN (40 wt.%). (**b**) Composites with n-AlN/μ-AlN (20/20 wt.%) and BN/ n-AlN/μ-AlN (20/10/10 wt.%).

For the dielectric characterization of the POx- and PA 12-based materials, permittivity measurements were performed in the frequency range of 0.1 to 5 kHz at room temperature. In the PA 12 samples, the real part of the permittivity increases with decreasing frequency (Figure 10). The permittivity of the SOE-free materials is in the range of 3 to 5, while the unfilled PA 12 network and all composites show similar values. Upon copolymerization with 15 and 30 wt.% of SOE, the real part of the permittivity of the polymer networks increases significantly. This effect is most pronounced in the low frequency range (<100 Hz), at which values of up to 13 are reached. 

**Figure 11:**
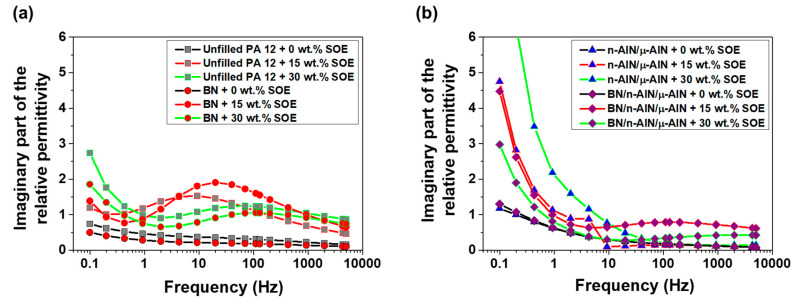
Imaginary part of the relative permittivity of the PA 12-based materials. (**a**) Unfilled PA 12 samples and composites with BN (40 wt.%). (**b**) Composites with n-AlN/μ-AlN (20/20 wt.%) and BN/ n-AlN/μ-AlN (20/10/10 wt.%).

**Figure 12:**
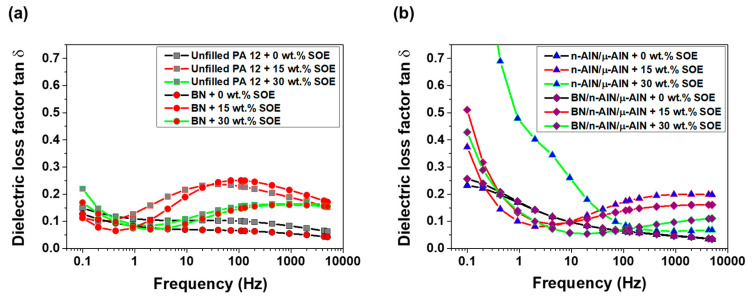
Dielectric loss factor of the PA 12-based materials. (**a**) Unfilled PA 12 samples and composites with BN (40 wt.%). (**b**) Composites with n-AlN/μ-AlN (20/20 wt.%) and BN/ n-AlN/μ-AlN (20/10/10 wt.%).

In the PA 12 series, the imaginary part of the relative permittivity of the SOE-free materials is the range of 0.15 to 1.3 and increases with decreasing frequency (Figure 11). Upon the addition of SOE, the imaginary part of the permittivity increases, in particular at frequencies below 10 Hz, which is indicative of interfacial polarization. Notably, this trend is most pronounced for the composites containing n-AlN and μ-AlN particles as fillers. These trends, which are observed for the imaginary part of the permittivity, are reproduced in the dielectric loss factor tan δ (Figure 12). In particular, for the composites containing n-AlN/μ-AlN and BN/n-AlN/μ-AlN as filler, the loss factor increases drastically at low frequencies. However, at approximately 50 Hz (industrial standard), low loss factors of 0.07 to 0.24 are observed (a detailed discussion and comparison of the loss factors of the PA 12- and POx-based materials is given hereinafter). 

One possible explanation for the increasing real and imaginary part of the permittivity upon copolymerization with the SOE is the volumetric expansion during curing. Due to expansion, the free volume of the polymer increases, and hence, the distances of the interaction zones between the fillers and the polymer matrix [28] and the polarizability of the materials are altered. In addition, the polarizability of the networks increases by the formation of more flexible polyester-*co*-polyether chains, which are formed during the ring-opening polymerization of the SOE groups.

**Figure 13:**
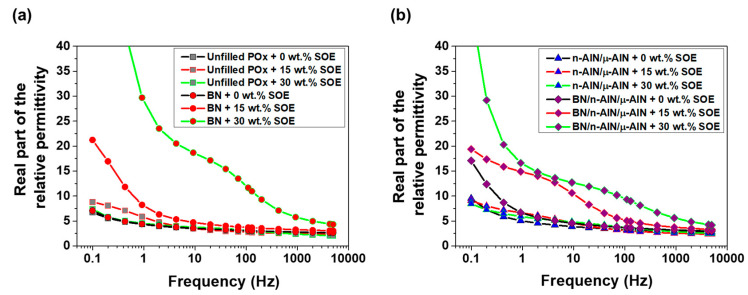
Real part of the relative permittivity of the POx-based materials. (**a**) Unfilled POx samples and composites with BN (40 wt.%). (**b**) Composites with n-AlN/μ-AlN (20/20 wt.%) and BN/n-AlN/μ-AlN (20/10/10 wt.%).

The real part of the permittivity of the POx-based, SOE-free networks is in the range of 2.5 to 17 and increases with decreasing frequency (Figure 13). The unfilled polymer and the composite containing n-AlN/μ-AlN particles as filler exhibit similar permittivities, and the addition of 15 or 30 wt.% of SOE only has a minor impact. In contrast, for the composites containing BN particles, the real part of the permittivity dramatically increases by the addition of 30 wt.% of the SOE. At the low frequency of 0.1 Hz, high values of above 100 (for the composite with 40 wt.% BN) and of 48 (for the composite with 20/10/10 wt.% BN/n-AlN/μ-AlN) are reached.

**Figure 14:**
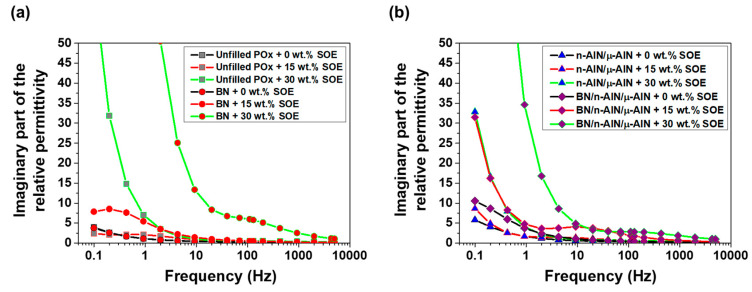
Imaginary part of the relative permittivity of the POx-based materials. (**a**) Unfilled POx samples and composites with BN (40 wt.%). (**b**) Composites with n-AlN/μ-AlN (20/20 wt.%) and BN/ n-AlN/μ-AlN (20/10/10 wt.%).

**Figure 15:**
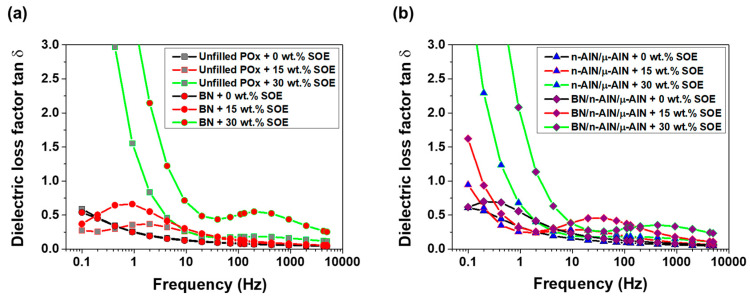
Dielectric loss factor of the POx-based materials. (**a**) Unfilled POx samples and composites with BN (40 wt.%). (**b**) Composites with n-AlN/μ-AlN (20/20 wt.%) and BN/ n-AlN/μ-AlN (20/10/10 wt.%).

In the POx series, the imaginary part of the permittivity of the SOE-free materials is in the range of 0.1 to 10 and increases with decreasing frequency (Figure 14). For the networks with a SOE content of 15 wt.%, a slight increase of the imaginary part of the permittivity was observed. The networks containing 30 wt.% of SOE show significantly increased values for the imaginary permittivity in the low-frequency range. This trend is most pronounced for the composites containing 40 wt.% of BN particles or 20/10/10 wt.% of BN/n-AlN/μ-AlN particles as filler. The observed trends for the imaginary part of the permittivity are observed for the loss factor in similar fashion (Figure 15). The materials containing 0 and 15 wt.% of the SOE show similar loss factors. Upon addition of 30 wt.% of the SOE, the values of the loss factor dramatically increase at frequencies below 10 Hz, and the insulating properties are deteriorated in this frequency range.

For the POx-based materials, it can be argued that the addition of 30 wt.% of the SOE as well as the addition of BN particles results in a high extension of interfacial polarization. Correspondingly, the permittivity and the loss factor of the materials strongly increase at low frequency.

In order to summarize, the loss factor tan δ of the PA 12- and POx-based networks was compared at a frequency of 40 Hz (Table 5 and Table 6). 

(i) Both types of unfilled, SOE-free networks (based on either PA 12 or POx) show the same low loss factor of 0.10; hence, the POx-based resins can be considered as a “green” alternative to those of fossil-based PA 12.

(ii) Due to the addition of fillers, the loss factor of the PA 12 networks is reduced to 0.07. The POx-based networks containing BN (40 wt.%) and n-AlN/μ-AlN (20/20 wt.%) are competitive to the PA 12-based materials and show low loss factors in the same range. 

(iii) The copolymerization of POx and PA 12 with 15 and 30 wt.% SOE, respectively, results in increased loss factors. Nevertheless, with a few exceptions (namely the POx-based composites containing BN and BN/n-AlN/μ-AlN as filler), all POx-based materials show low loss factors in the range of fossil-based polyamides [24].

## 4. Conclusions

The aim of this study was the preparation of (nano-)dielectrics with reduced volumetric shrinkage from renewable resources. The copoly(2-oxazoline), namely pNonOx_80_-*stat*-pDec^=^Ox_20_, was synthesized by the energy-efficient microwave-assisted cationic ring-opening copolymerization of two 2-oxazoline monomers, which were derived from renewable resources (fatty acids) in a solvent-free synthesis. The dielectric and physicochemical properties of the POx were compared with those of fossil-based, commercially available PA 12.

In order to reduce the shrinkage during curing, different amounts of a spiroorthoester SOE containing C=C double bonds (named SOE-allyl) were added as expanding monomers. In addition, composites containing 40 wt.% of nano-sized and micron-sized AlN particles and submicron-sized BN particles (in different combinations of the fillers) were prepared. For crosslinking of the polymers, a thermally triggered dual-cure system was developed, comprising the radical-mediated crosslinking of the polymers and the SOE-allyl as well as the cationic ring-opening polymerization of the SOE groups upon volumetric expansion. 

Upon copolymerization with the SOE, *relative* expansion (RE) in the range of +0.46% to +2.48% for the PA 12-based materials and in the range of +1.39% to +7.50% for the POx-based materials was achieved. The thermal diffusivity of the polymers was significantly increased by the addition of 40 wt.% of the inorganic fillers; the thermal diffusivity was highest in the composites containing 40 wt.% of BN. The thermal diffusivity decreased upon the addition of the SOE due to volumetric expansion and the reduced density of the polymers. 

The dielectric characterization of the materials revealed an increase of the permittivity and loss factor tan δ upon the addition of the SOE-allyl in the low-frequency range due to interfacial polarization and the formation of more flexible polyester-*co*-polyether chains. At the industrial frequency standard of 50 Hz, the POx-based materials showed low loss factors in a similar range to that of the PA-based networks. Hence, the “green” POx networks exhibit competitive dielectric performance compared to the fossil-based PA 12 networks. 

## Figures and Tables

**Table 1:** Densities of the POx-based materials.

SOE (wt.%)	Unfilled ρ (g∙cm^−3^)	BN, 40 wt.% ρ (g∙cm^−3^)	n-AlN/μ-AlN, 20/20 wt.% ρ (g∙cm^−3^)	BN/n-AlN/μ-AlN, 20/10/10 wt.% ρ (g∙cm^−3^)
0	1.05	1.23	1.29	1.29
15	1.04	1.21	1.24	1.23
30	1.01	1.17	1.19	1.19

**Table 2:** Densities of the PA 12-based materials.

SOE (wt.%)	Unfilled ρ (g∙cm^−3^)	BN, 40 wt.% ρ (g∙cm^−3^)	n-AlN/μ-AlN, 20/20 wt.% ρ (g∙cm^−3^)	BN/n-AlN/μ-AlN, 20/10/10 wt.% ρ (g∙cm^−3^)
0	1.02	1.21	1.26	1.28
15	1.01	1.20	1.25	1.27
30	0.99	1.19	1.24	1.26

**Table 3:** Relative expansions (REs) of the POx-based materials.

SOE (wt.%)	RE (%)			
	No Filler	BN 40 wt.%	n-AlN/μ-AlN 20/20 wt.%	BN/n-AlN/μ-AlN 20/10/10 wt.%
0	0	0	0	0
15	1.54	1.39	3.70	4.97
30	4.39	4.84	7.69	7.50

**Table 4:** Relative expansions (REs) of the PA 12-based materials.

SOE (wt %)	RE (%)			
	No Filler	BN 40 wt.%	n-AlN/μ-AlN 20/20 wt.%	BN/n-AlN/μ-AlN 20/10/10 wt.%
0	0	0	0	0
15	0.98	0.84	0.46	0.84
30	2.48	1.41	1.92	1.60

**Table 5:** Dielectric loss factor tan δ of the PA 12-based materials at 40 Hz.

SOE (wt.%)	tan δ			
	No Filler	BN 40 wt.%	n-AlN/μ-AlN 20/20 wt.%	BN/n-AlN/μ-AlN 20/10/10 wt.%
0	0.10	0.07	0.07	0.07
15	0.24	0.24	0.15	0.12
30	0.14	0.13	0.13	0.06

**Table 6:** Dielectric loss factor tan δ of the POx-based materials at 40 Hz.

SOE (wt.%)	tan δ			
	No Filler	BN 40 wt.%	n-AlN/μ-AlN 20/20 wt.%	BN/n-AlN/μ-AlN 20/10/10 wt.%
0	0.10	0.09	0.11	0.16
15	0.16	0.18	0.25	0.46
30	0.17	0.44	0.18	0.26

## Data Availability

The data are not yet publicly available due to the performance of on-going studies of the scientific findings; they are available on request from the corresponding author.
